# Three novel leaderless bacteriocins have antimicrobial activity against gram-positive bacteria to serve as promising food biopreservative

**DOI:** 10.1186/s12934-022-01912-3

**Published:** 2022-09-19

**Authors:** Xiaofeng Zhang, Nie Xin, Zhaolu Zhu, Xudong Li, Dadong Dai, Chunmei Pan, Donghai Peng, Ming Sun

**Affiliations:** 1 State Key Laboratory of Agricultural Microbiology, College of Life Science and Technology, Huazhong Agricultural University, Wuhan, 430070 People’s Republic of China; 2 College of Food and Biological Engineering, Henan University of Animal Husbandry and Economy, Zhengzhou, China

**Keywords:** Leaderless bacteriocin, Biopreservative, *Bacillus thuringiensis*, Thucin A1

## Abstract

**Background:**

Due to the detrimental effects of chemical preservatives, there has been an increasing demand for safer, healthier and natural bio-preservatives. Bacteriocins have attracted increasing interest because of their potential as natural bio-preservatives.

**Results:**

We screened a large number of *Bacillus thuringiensis* strains and isolated one strain (*B. thuringiensis* P86) with antimicrobial activity against several foodborne pathogens. Three novel leaderless bacteriocins, including thucin A1, thucin A2 and thucin A3, were purified and identified from the culture supernatant of *B. thuringiensis* P86, whose molecular masses were 5552.02, 5578.07 and 5609.06 Da, respectively. Thucin A1 was then selected as a representative to be tested, and it exhibited potent inhibitory activity against all tested gram-positive bacteria. More importantly, thucin A1 showed stronger antimicrobial activity than nisin A against two important foodborne pathogens *Bacillus cereus* and *Listeria monocytogenes*. In addition, thucin A1 exhibited strong acid–base adaptability (pH 2–11), high endurance to heat, good stability to trypsin and pepsin, no hemolysis activity and cytotoxicity, and could effectively inhibit or eliminate *Bacillus cereus* and *Listeria monocytogenes* in skim milk.

**Conclusions:**

Our findings indicate that these novel leaderless bacteriocins are potentially promising food biopreservatives.

**Supplementary Information:**

The online version contains supplementary material available at 10.1186/s12934-022-01912-3.

## Background

Food pathogenic or spoilage bacteria pose serious threats to food quality and human health [1–3]. Chemical preservatives generally have certain detrimental effects, and therefore consumers show increasing demand for safer, healthier and natural biopreservatives [4]. Bacteriocins are a diverse group of peptides or proteins ribosomally synthesized by bacteria, which can inhibit or kill other bacteria and have a great potential to be used as biopreservatives [5, 6]. Generally, bacteriocins produced by gram-positive bacteria can be divided into three major classes: class I that consists of ribosomally synthesized and post-translationally modified peptides (RiPPs) that function as bacteriocins, such as lantibiotics, lipolanthines, linear azol(in)e-containing bacteriocins, thiopeptides, bottromycins, sactibiotics, lasso peptides, glycocins, head-to-tail cyclized bacteriocins; class II that consists of small unmodified peptides including YGNG-motif containing bacteriocins, two-peptide bacteriocins, leaderless bacteriocins and other linear bacteriocins; and class III that consists of large heat-labile bacteriocins, such as tailocins [7]. Bacteriocins have several advantages to be applied in food preservation, such as safety, non-toxicity, heat stability, easy digestion by the human gastrointestinal tract, effect against food spoilage or pathogenic bacteria at low concentrations, action on the bacterial cytoplasmic membrane and no cross resistance with antibiotics [8]. Therefore, bacteriocins have been extensively studied in terms of their potential use in enhancing food safety and quality [9]. So far, only one bacteriocin, nisin A, which is produced by certain strains of *Lactococcus lactis*, has been widely used as a food biopreservative [10]. However, nisin A can only be applied under acidic conditions since its antimicrobial activity will be lost under neutral or alkaline conditions [11]. Hence, it is critical and urgent to discover new bacteriocins to prevent the risk of pathogenic or spoilage bacteria in food.

*Bacillus thuringiensis* is a ubiquitous gram-positive, spore-forming bacterium that produces parasporal crystals during the stationary phase of its growth cycle [12]. Recent studies have demonstrated that *B. thuringiensis* is a prominent producer of bacteriocins and contains various types of novel bacteriocins that have not been functionally characterized [5, 13]. So far, many bacteriocins have been identified in *B. thuringiensis*, such as thuricin 4AJ1 (not fully characterized), thuricin 7 (not fully characterized), thuricin Bn1 (sactibiotic), BtCspB (not fully characterized), thurincin H (sactibiotic), thuricin CD (two-peptide sactibiotic) and thuricin Z (sactibiotic) [14–19].

In this study, in order to discover novel bacteriocins against food pathogenic bacteria, a large number of *B. thuringiensis* strains from soil samples were tested for their antimicrobial activities against two important foodborne pathogens *Bacillus cereus* ATCC 14579 and *Listeria monocytogenes* LM201. As a result, three novel leaderless bacteriocins with antimicrobial activity against gram-positive bacteria produced by the strain *B. thuringiensis* P86, including thucin A1, thucin A2 and thucin A3, were purified and characterized. In addition, thucin A1 was selected as a representative and its antimicrobial activity as well as stability to pH, heat and protease were studied. Finally, the safety of thucin A1 to be used as a food preservative was evaluated, and its potential to be applied in food preservation was determined by evaluating its antimicrobial effect on foodborne pathogens in skim milk.

## Results

### Screening for producers of broad-spectrum antimicrobial bacteriocins

**Fig. 1:**
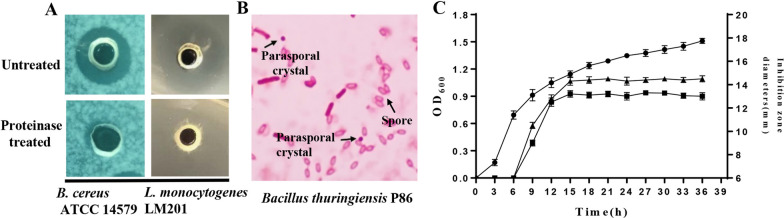
Detection of antimicrobial activities of *B. thuringiensis* P86. **A** The antimicrobial substances from *B. thuringiensis* P86 were treated with mixed protease solution. **B** Spore and parasporal crystal of *B. thuringiensis* P86. **C** Growth kinetics of *B. thuringiensis* P86 in LB medium and the production kinetics of antimicrobial substances. The optical density of the *B. thuringiensis* P86 culture was measured at 600 nm (●). The antimicrobial substance concentration was expressed as inhibition zone diameter. Indictor strain was *B. cereus* ATCC 14579 (■), *L. monocytogenes* LM201 (▲)

A total of 100 *B. thuringiensis* strains from soil samples were tested for their antimicrobial activity against two important food pathogenic bacteria *B. cereus* ATCC 14579 and *L. monocytogenes* LM201 to screen the potential bacteriocin producers. As a result, the culture supernatant of 12 strains exhibited antimicrobial activity against both two indicator bacteria, particularly the *B. thuringiensis* P86 strain, which exhibited the strongest antimicrobial activity. As *B. thuringiensis* P86 lost its antimicrobial activity after the culture supernatant was treated by mixed proteinase solution (Fig. 1A), we suspected that the antimicrobial substance was bacteriocin, and thus chose *B. thuringiensis* P86 for further analysis. Spores and parasporal crystals could be clearly observed when *B. thuringiensis* P86 was incubated on LB agar plates for 48 h at 30 °C (Fig. 1B). The antimicrobial substances were produced during the exponential growth phase and the maximum antimicrobial substance production was recorded after 15 h (Fig. 1C). Moreover, the antimicrobial activity against *B. cereus* ATCC 14579 is significant different from that of *L. monocytogenes* LM201 after 15 h (p < 0.05).

### Identification and characterization of a putative bacteriocin gene cluster in B. thuringiensis P86

**Fig. 2:**
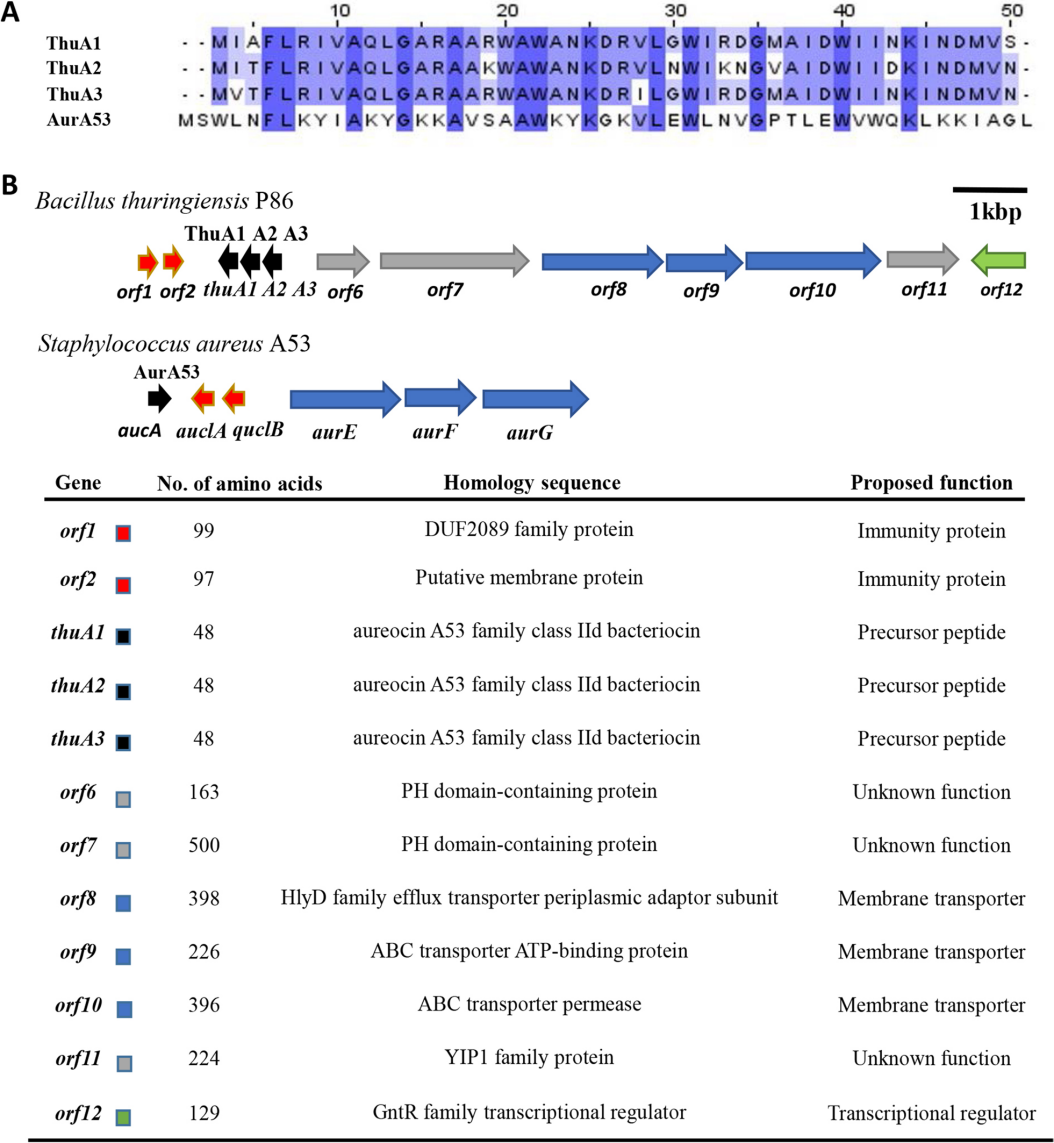
Predicted amino acid sequences of precursor peptides and biosynthetic gene clusters of thucin A. **A** Sequence alignment of the precursor peptides with their analogues. **B** Gene clusters of thucin A and the analogues. Characteristics of predicted proteins encoded by putative genes in the thucin A gene cluster. Predicted proteins are colored according to their predicted functions

To search for the gene cluster responsible for the synthesis of the antimicrobial substances, we sequenced the genome of *B. thuringiensis* P86 and analyzed all putative bacteriocin biosynthetic gene clusters with the BAGEL 4.0 online software. As a result, only one putative bacteriocin biosynthetic gene cluster, which was termed as the thucin A gene cluster, could potentially synthesize bacteriocins (Fig. 2B). This gene cluster comprised 12 genes, including three tandem and highly homologous structural genes (*thuA1*, *thuA2* and *thuA3*) encoding putative bacteriocins, two genes (*orf1* and *orf2*) encoding the DUF2089 family protein and putative membrane protein possibly involved in immunity, two genes (*orf6* and *orf7*) encoding the PH domain-containing protein with unknown function, three genes (*orf8*, *orf9* and *orf10*) encoding the HlyD family membrane transporter protein or ABC transporter-related protein that may contribute to the secretion of bacteriocins, one gene (*orf11*) encoding a YIP1 family protein with unknown function, and one gene (*orf12*) encoding a GntR family transcriptional regulator protein. The amino acid sequences of the three precursor peptides were nearly identical (identities above 83%) and their theoretical monoisotopic mass was 5523.95, 5550.01 and 5580.97 Da, respectively (Fig. 2A). Precursor peptide (ThuA1, ThuA2 and ThuA3) analysis revealed that their closest precursor peptide is AurA53, the precursor of identified leaderless bacteriocin aureocin A53 [20]. AurA53 showed low homologies with ThuA1, ThuA2 and ThuA3 (33%, 30% and 29%, respectively).

### Purification and identification of antimicrobial substances

**Fig.3:**
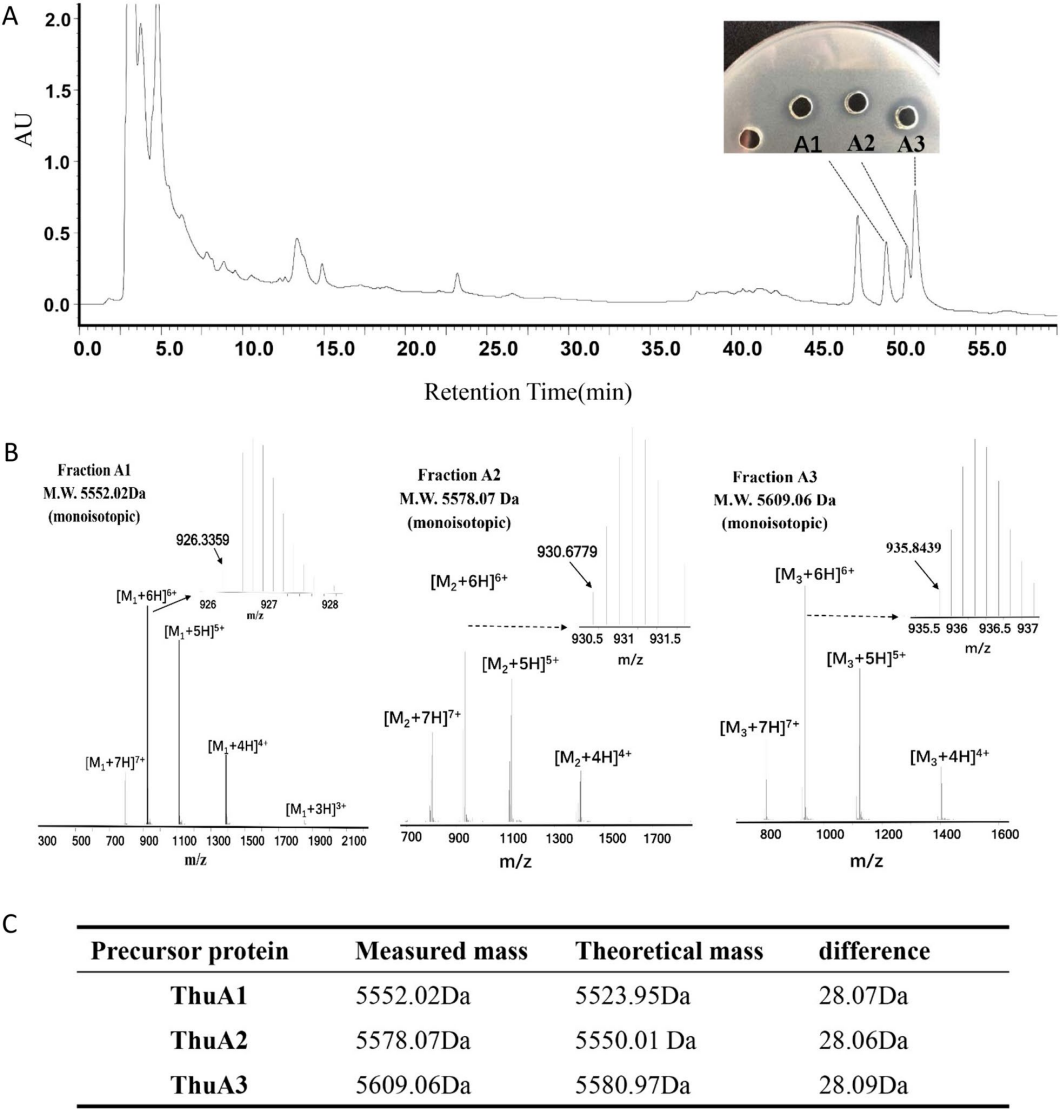
Purification and MS analysis of antimicrobial substance. **A** HPLC analysis of crude extracts of antimicrobial substance. The fractions were monitored at 210 nm and manually collected. **B** Liquid chromatography-mass spectrometry (LC–MS) analysis of fractions A1, A2, A3 molecular mass. **C** Comparison of the measured mass of antimicrobial fractions and the theoretical mass of precursor peptides

*B. thuringiensis* P86 was cultured in LB broth for 15 h and the antimicrobial substances in the culture supernatant were concentrated by Amberlite XAD-7 HP resin. Then, the antimicrobial crude extract was loaded onto an Agilent TC-C18 column and separated by HPLC. As shown in Fig. 3A, only three fractions (A1, A2 and A3), which corresponded to three peaks with retention time of 49.5, 50.7 and 51.4 min, are active against *B. cereus* ATCC 14,579. Each antimicrobial fraction was collected and subjected to LC–MS analysis. The measured monoisotopic mass of fraction A1, A2 and A3 is 5552.02 Da (m/z 926.3359), 5578.07 Da (m/z 930.6779), 5609.06 Da (m/z 935.8439), respectively (Fig. 3B), which are all approximately 28 Da higher than the theoretical monoisotopic mass of ThuA1, ThuA2 and ThuA3, indicating that the first amino acid of the three precursor peptides was a formylated methionine (Fig. 3C). The mature peptides of ThuA1, ThuA2 and ThuA3 were designated as thucin A1, thucin A2 and thucin A3, respectively.

**Fig. 4:**
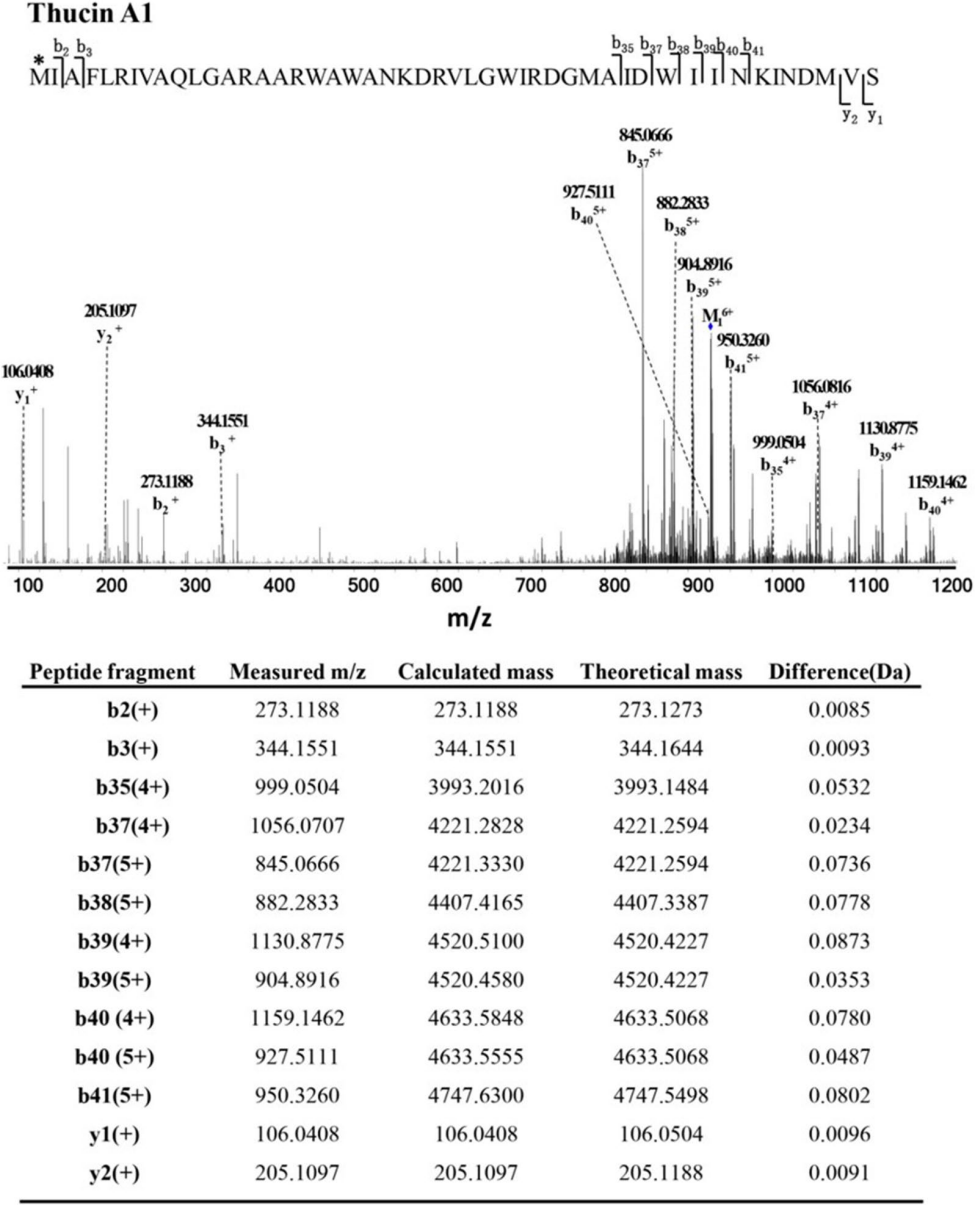
Proposed primary structure of thucin A1 and LC–MS/MS analysis of fraction A1. Fragment ions are indicated. “*” indicates that the N-terminal amino acid, methionine, was formylated

To investigate the detailed sequence information of the antimicrobial fractions, fractions A1, A2 and A3 were further analyzed using LC–MS/MS. In the MS/MS spectrum of fraction A1, the measured molecular mass of all the marked fragment ions was consistent with the theoretical molecular mass of thucin A1 fragments (Fig. 4), confirming that fraction A1 was the mature peptide thucin A1. The same results were observed for fraction A2 and A3 as shown in Additional file 1: Figs. S1 and S2. According to these results, the fragments of fraction A1, A2 and A3 could correspond to the fragments of mature peptides thucin A1, thucin A2 and thucin A3. Therefore, thucin A1, thucin A2 and thucin A3 were regarded as novel types of leaderless bacteriocins.

### Antimicrobial activity of thucin A1

As thucin A1, thucin A2 and thucin A3 had nearly identical amino acid sequences and only the purity of thucin A1 was obtained, thucin A1 was chosen as the representative to be tested against a series of indicator bacteria. Thucin A1 showed antimicrobial activities against all of the gram-positive bacteria, including *B. cereus*, *L. monocytogenes*, *B. thuringiensis, S. aureus. S. succinus*, *B. subtilis*, *B. pumilus*, *B. firmus*, *B. simplex*, *L. fusiformis*, *R. hodococcus* sp., *G. arilaitensis* and *B. siamensis*, but not against any of the tested gram-negative bacteria (Table 1). In addition, we compared the antimicrobial activity of thucin A1 and nisin A. The results showed that the antibacterial activity of thucin A1 against *B. cereus* ATCC 14579, *B. cereus* UW85, *L. monocytogenes* LM201 and *L. monocytogenes* LM605 was 2–8 folds that of nisin A, and the antibacterial activity of thucin A1 against *B. thuringiensis* BMB171, *B. pumilus* SCG1, *B. firmus* DS-1 and *B. simplex* SYY08 was comparable to that of nisin A. These results demonstrated that thucin A1 has antimicrobial activity against gram-positive bacteria, and the antimicrobial activity against the bacteria from the *Bacillus* and *Listeria* genera was stronger than that against bacteria from other genera.

**Table 1:** Antimicrobial activities of purified thucin A1 and nisin A

Indicator strain (reference)^a^	Culture medium^b^	MIC (μM)^c^	
		Thucin A1	Nisin A
Gram-positive bacteria			
* Bacillus cereus* ATCC 14579	LB	1.88	7.50
* Bacillus cereus* UW85 [21]	LB	1.88	7.50
* Listeria monocytogenes* LM201 [22]	LB	0.47	3.75
* Listeria monocytogenes* LM605 [23]	LB	0.47	3.75
* Bacillus thuringiensis* BMB171[24]	LB	1.88	1.88
* Bacillus thuringiensis* P86	LB	7.5	3.75
* Staphylococcus aureus* ATCC 43300	LB	15	3.75
* Staphylococcus succinus* SYY04	LB	15	3.75
* Bacillus subtilis* Bsn5 [25]	LB	3.75	0.94
* Bacillus subtilis* 168 [26]	LB	3.75	1.88
* Bacillus pumilus* SCG1 [13]	LB	3.75	3.75
* Bacillus firmus* DS-1 [27]	LB	3.75	3.75
* Bacillus simplex* SYY08	LB	1.88	1.88
* Lysinibacillus fusiformis* ZZY15-1	LB	7.5	1.88
* Rhodococcus* sp*.* ZCH08	LB	15	7.50
* Glutamicibacter arilaitensis* HCX33	LB	7.5	0.94
* Bacillus siamensis* HCX05	LB	3.75	1.88
Gram-negative bacteria			
* Escherichia coli* ATCC25922	LB	–	–
* Escherichia coli* DH5α	LB	–	–
* Salmonella paratyphi* CMCC 50,093	LB	–	–
* Erwinia herbicola* LS005[13]	LB	–	–
* Pseudomonas putiida* Pri3[13]	LB	–	–
* Stenotrophomonas maltophilia* SYY20	LB	–	–

^a^See references for the sources of the marked strains. ATCC, American Type Culture Collection. CMCC, China Medical Culture Collection. Unmarked strains were isolated from the air in Zhengzhou city, Henan Province in China, and identified by 16S rRNA gene sequence analysis according to the reported method [5]

^b^LB, Luria broth

^c^The highest concentration of thucin A1 and nisin A was 60 μM. "-" indicates the absence of activity against indicator strains even at the highest concentration of the indicated peptides

### Comparison of the stability of thucin A1 and nisin A to pH, temperature and protease

**Fig. 5:**
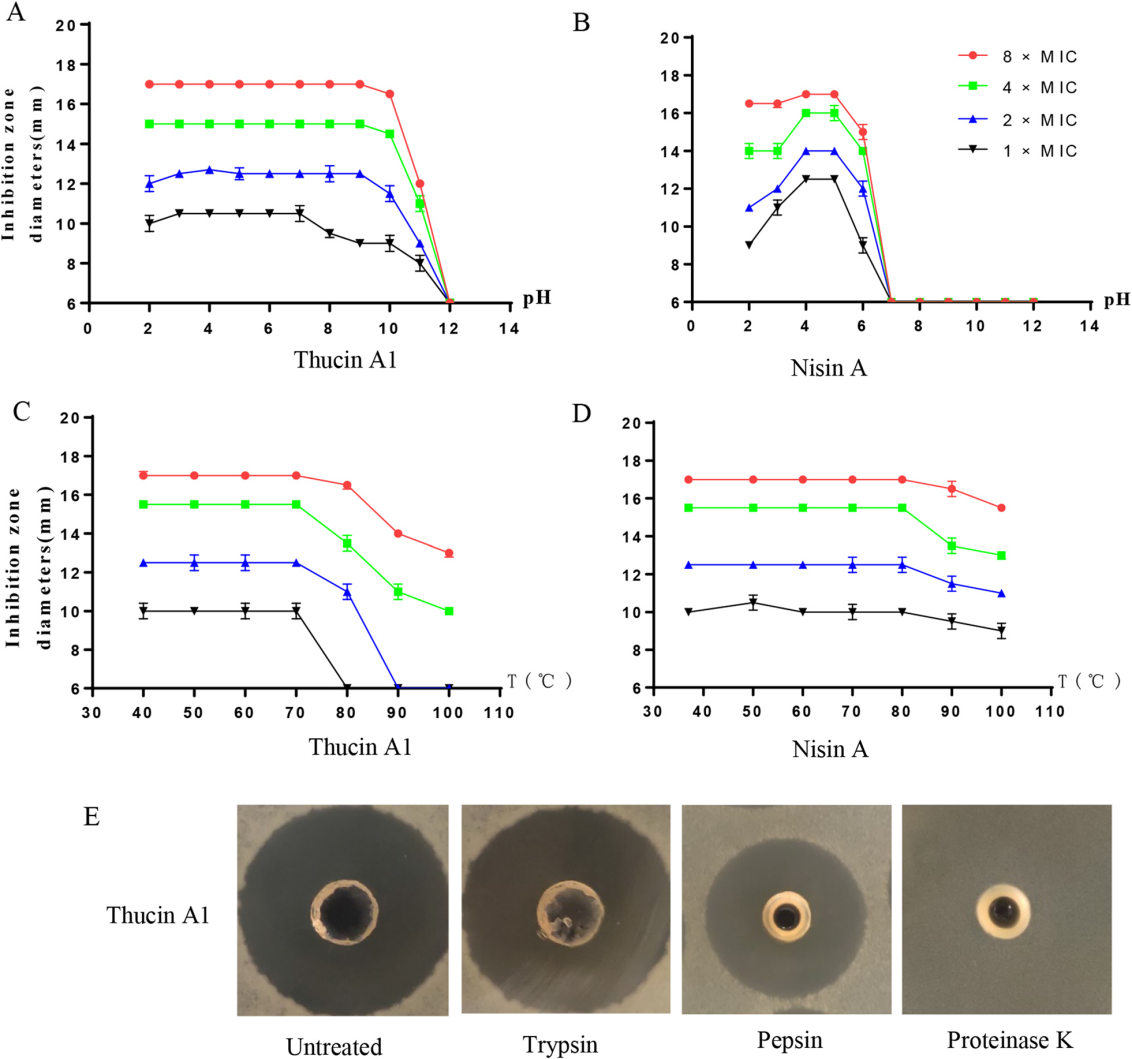
Stability comparison of thucin A1 and nisin A. The indicator strain was *B. cereus* ATCC 14579 and untreated bacteriocin solutions were used as the control. **A** Stability of thucin A1 and nisin A to pH. **B** Stability of thucin A1 and nisin A to temperature. **C** Stability of thucin A1 to protease treatment

The sensitivity of thucin A1 and nisin A to pH was tested with the agar diffusion method. The HPLC-purified thucin A1 was tested for sensitivity to pH. As shown in Fig. 5A, thucin A1 had activity at different concentrations (1× , 2× , 4× , 8× MIC) and pH ranging from 2.0 to 11.0, and at concentrations above 1× MIC, the activity was extremely stable at pH 2–9. The residual antimicrobial activity was greatly decreased when the pH was greater than 10. In parallel experiments, nisin A at all concentrations (1× , 2× , 4× , 8× MIC) showed no antimicrobial activity at pH > 6 (Fig. 5B). Therefore, thucin A1 has certain advantages over nisin A in terms of antimicrobial activity at pH > 6, and therefore higher stability in a wider range of pH values.

The sensitivity of thucin A1 and nisin A to temperature is presented in Fig. 5C and D. Above 70 °C, the antimicrobial activity of thucin A1 decreased with increasing temperature. In parallel experiments, nisin A at all concentrations could resist the temperature of 100 °C, although the activity was somewhat decreased above 80 °C. These results indicated that thucin A1 at low concentrations is less resistant to heat than nisin A, while its heat resistance is generally comparable to that of nisin A at high concentrations.

Thucin A1 (4× MIC) could retain its full antimicrobial activity after treatment with trypsin, partial antimicrobial activity after treatment with pepsin, but completely lost its antimicrobial activity after digestion with proteinase K (Fig. 5E). Under the same conditions, nisin A (4 × MIC) completely lost its antimicrobial activity after digestion with trypsin, pepsin and proteinase K (data not shown).

### Bactericidal activity of thucin A1

**Fig. 6:**
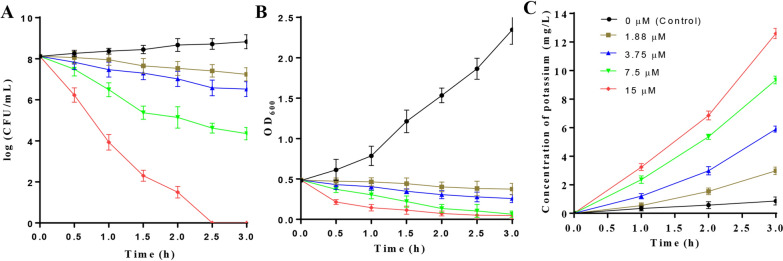
Thucin A1 exerts bactericidal activity. **A** Effect of thucin A1 on the number of viable cells of *B. cereus* ATCC 14579 culture. **B** Effect of thucin A1 on the optical density of *B. cereus* ATCC 14579 culture. **C** Effect of thucin A1 on amount of potassium released from *B. cereus* ATCC 14579 cells. All experiments were performed in triplicate and the data are shown as mean ± SD

The mode of action of thucin A1 was deduced by incubation of different concentrations (0, 1.88, 3.75, 7.5, 15 μM) of HPLC-purified thucin A1 in the culture of the *B. cereus* ATCC 14579 (OD_600_ ~ 0.5). The number of viable cells and the OD_600_ of the culture were monitored at different time points. As shown in Fig. 6A and ,B, the number of viable cells and OD_600_ value significantly decreased after 1.0 h when the concentration of thucin A1 was higher than 3.75 μM, relative to that of the control (p ≤ 0.05). Moreover, no viable cells were observed after 2.5 h at a high concentration of thucin A1 (15 μM). These results indicated that thucin A1 has bactericidal activity against sensitive strains. In addition, the cell membrane integrity of *B. cereus* after incubation with thucin A1 was evaluated by potassium efflux assay. As shown in Fig. 6C, after treatment with different concentration thucin A1 (1.88, 3.75, 7.5, 15 μM) for 3 h, the concentrations of potassium in the supernatants increased significantly relative to that of the control (p ≤ 0.01), indicating the cell membrane of *B. cereus* was damaged. This result also supports that thucin A1 has bactericidal activity against sensitive strains.

### Non-hemolytic activity and non-cytotoxicity of thucin A1

**Fig. 7:**
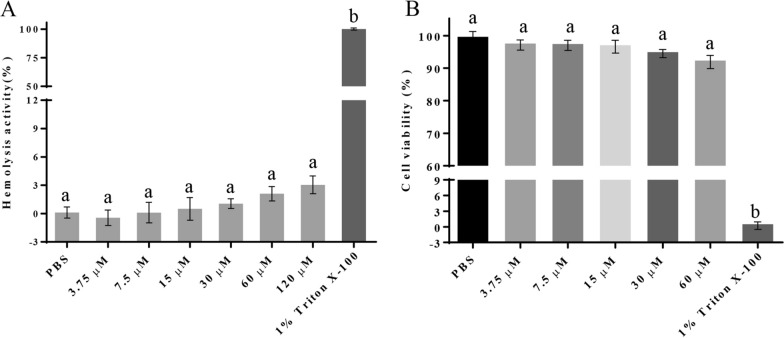
The hemolysis activity of thucin A1 (**A**) and the cell viability of thucin A1 (**B**). Significantly different values are denoted by different lowercase letters (P < 0.05). All experiments were performed in triplicate and the data are shown as mean ± SD

To assess the safety of thucin A1, the hemolytic activity assay and cytotoxicity assay of thucin A1 were performed. we examined the hemolysis activity of thucin A1 at different concentrations (3.75, 7.5, 15, 30, 60 and 120 μM) in animal eukaryotic cells. As shown in Fig. 7A, all the test results were significantly different from those of the positive control (1% Triton X-100) (*p* < 0.01). Moreover, when the concentration of thucin A1 was lower than or equal to 30 μM, no hemolysis of red blood cells was observed, and the results were not significantly different from those obtained with the phosphate-buffered saline (PBS) buffer (*p* > 0.1). In addition, we measured the cell viability of HeLa cells (human cervical cancer cells) containing different concentrations thucin A1 (3.75, 7.5, 15, 30 and 60 μM). As shown in Fig. 7B, all the test results were not significantly different from those of the negative controls (*p* > 0.1) even the concentration of thucin A1 is up to 60 μM. These results indicated that thucin A1 does not cause hemolysis and has not cytotoxicity even at concentrations much higher than the MIC values against sensitive strains.

### Application of thucin A1 to the preservation of skim milk

**Fig. 8:**
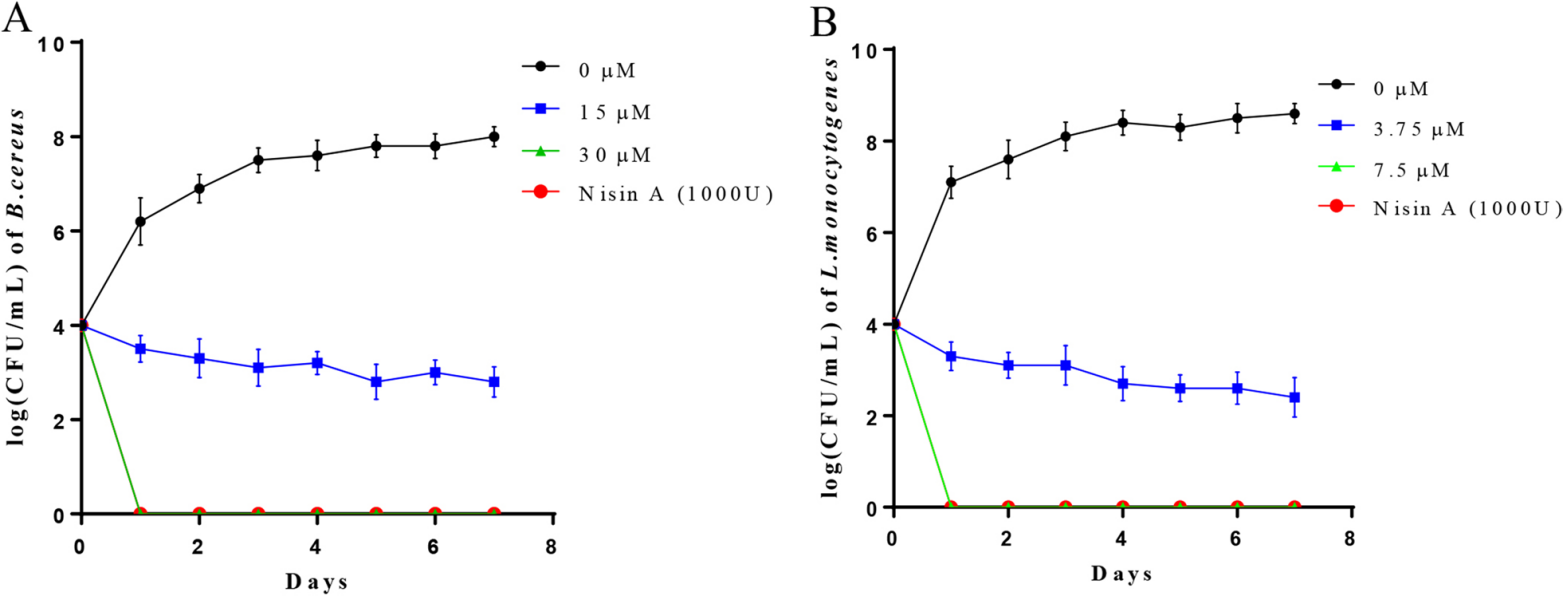
Effect of thucin A1 on the growth of pathogens in skim milk. **A** The skim milk samples with *B. cereus* ATCC14579 were stored at 25 °C. **B** The skim milk samples with *L. monocytogenes* LM201 were stored at 25 °C. Nisin A (1000U) and ddH_2_O were used as the positive and negative controls, respectively. The data are shown as mean ± SD

The activity of thucin A1 to suppress *B. cereus* and *L. monocytogenes* in skim milk samples was evaluated. As shown in Fig. 8, The growth of *B. cereus* and *L. monocytogenes* was markedly inhibited during the storage period when the concentration of thucin A1 was 15 μM and 3.75 μM, and the difference was 5.2 ± 0.15 and 6.2 ± 0.20 log CFU/mL relative to the control on day 7 (p < 0.01). At the concentration of 30 μM, thucin A1 completely killed *B. cereus* and *L. monocytogenes* on the first day. Nisin A (1000 U) was used as the positive control, and *B. cereus* and *L. monocytogenes* all died on the first day.

## Discussion

This study aims to screen and identify novel bacteriocins against two important food pathogenic bacteria *B. cereus* ATCC 14579 and *L. monocytogenes* LM201. To this end, 100 *B. thuringiensis* strains from soil samples were tested for their antibacterial activity to identify potential bacteriocin producers. The *B. thuringiensis* P86 strain, which is the producer of three novel leaderless bacteriocins, was selected for further study. Leaderless bacteriocins represent a class of antimicrobial peptides derived from ribosome and are characterized by the absence of an N-terminal leader peptide. These bacteriocins do not undergo any post-translational modification or processing and become active soon after translation [23]. As a class of bacteriocins, they hold great potential to be used as a food biopreservative.

Enterocin L50, the first reported leaderless bacteriocin, is composed of two peptides (enterocin L50A and L50B) with synergistic activity [28]. To date, a total of 17 leaderless bacteriocins have been purified and identified, including enterocin L50, enterocin EJ97 and enterocin DD14 (same as enterocin MR10 or enterocin 7) from *Enterococcus faecalis* [28–31], enterocin Q and enterocin K1 from *Enterococcus fa*ecium [32, 33], aureocin A53 and aureocin A70 from *Staphylococcus aureus* [20, 34], weisselicin Y and weissellicin M from *Weissella hellenica* [35], lsbB, lacticin Q, lacticin Z and lactolisterin BU from *Lc. Lactis* [36–39], BHT-B from *Streptococcus rattus* [40], epidermicin NI01 from *Staphylococcus epidermidis* [41], garvicin KS from *Lactococcus garvieae* [23], and toyoncin from *Bacillus toyonensis* [42]. These leaderless bacteriocins are derived from bacterica of different species or genera, but there has been no report about the leaderless bacteriocins derived from *B. thuringiensis*. Therefore, this study is probably the first report of three novel leaderless bacteriocins (thucin A1, thucin A2 and thucin A3) produced by *B. thuringiensis* with antimicrobial activity against gram-positive bacteria.

Whole-genome sequencing and DNA analysis of *B. thuringiensis* P86 showed the thucin A biosynthetic gene cluster (Fig. 2B). This gene cluster comprised three tandem genes encoding the thucin A1 thucin A2 and thucin A3 precursors, two genes encoding proteins possibly involved in immunity, three genes encoding proteins for the secretion of bacteriocins, one gene encoding a transcriptional regulator protein, and other three genes encoding proteins with unknown functions. Sequence homology analysis revealed that the thucin A biosynthetic gene cluster is present in *B. bingmayongensis*, *B. mycoides*, *B. cereus* and other species, suggesting that the thucin A gene cluster has undergone horizontal transfer across the genus during evolution.

We observed that the theoretical masses of all putative bacteriocins determined based on the DNA sequence had a difference of ~ 28 Da from the peptide masses obtained by MS, indicating formylation of the first methionine residue of the peptides. These results were further verified by LC–MS/MS analysis. Most reported leaderless bacteriocins have formylated methionine at their N-terminal, such as aureocin A53, lacticin Q, lacticin Z, epidermicin NI01, lactolisterin BU, BHT-B, weisselicin Y, weissellicin M, garvicin KS, enterocin 7 and toyoncin. Formylation of the first methionine residue of the peptides is a feature that can distinguish leaderless bacteriocins from bacteriocins with leader sequences [43]. However, the formylation of methionine at the N-terminal may have no significant effect on the bioactivity of leaderless bacteriocins. The effect of formylation on the antimicrobial activity of bacteriocins can be further studied by artificial synthesis in subsequent research.

Previous reports have demonstrated that most leaderless bacteriocins have antimicrobial activity against food pathogenic bacteria *L. monocytogenes,* such as lacticin Q, garvicin KS, aureocin A70, weisselicin Y and weissellicin M [23, 34, 35, 37]. Here, thucin A1 exhibited antimicrobial activity against all gram-positive bacteria, including *B. cereus, L. monocytogenes, B. thuringiensis, S. aureus, B. subtilis*, *B. pumilus*, *B. firmus*, and the bacteria isolated in air (*S. succinus*, *B. simplex*, *L. fusiformis*, *R. hodococcus sp.*, *G. arilaitensis* and *B. siamensis*), which are important causes of food spoilage because they are more likely to contaminate food during food processing, transport and storage. These results indicate thucin A1 has antimicrobial activity gram-positive bacteria. Moreover, the antimicrobial activity of thucin A1 against *B. thuringiensis* BMB171 is fourfold higher than that against *B. thuringiensis* P86 (thucin A1 producer strain), indicating the presence of immunity proteins in thucin A gene cluster. In addition, thucin A1 exhibited a stronger antimicrobial activity against food borne pathogenic bacteria than nisin A, particularly against *B. cereus* and *L. monocytogenes*. *B. cereus* may not only cause food spoilage, but also act as pathogenic bacteria to cause inflammatory disease, respiratory infection, systemic infection and intestinal disorder by gaining access to human tissues [4]. *L. monocytogenes* is widely present in water, soil, meat products and vegetables and can grow during food processing, transport and refrigeration. They can cause meningitis, sepsis, gastroenteritis and other diseases in humans and animals [44, 45]. Currently, nisin A is widely used as a food biopreservative in food industry. These results suggest thucin A1 has great potential to be used for inhibiting food pathogenic bacteria, and may serve as a promising supplement of nisin A as a food biopreservative.

**Fig. 9:**
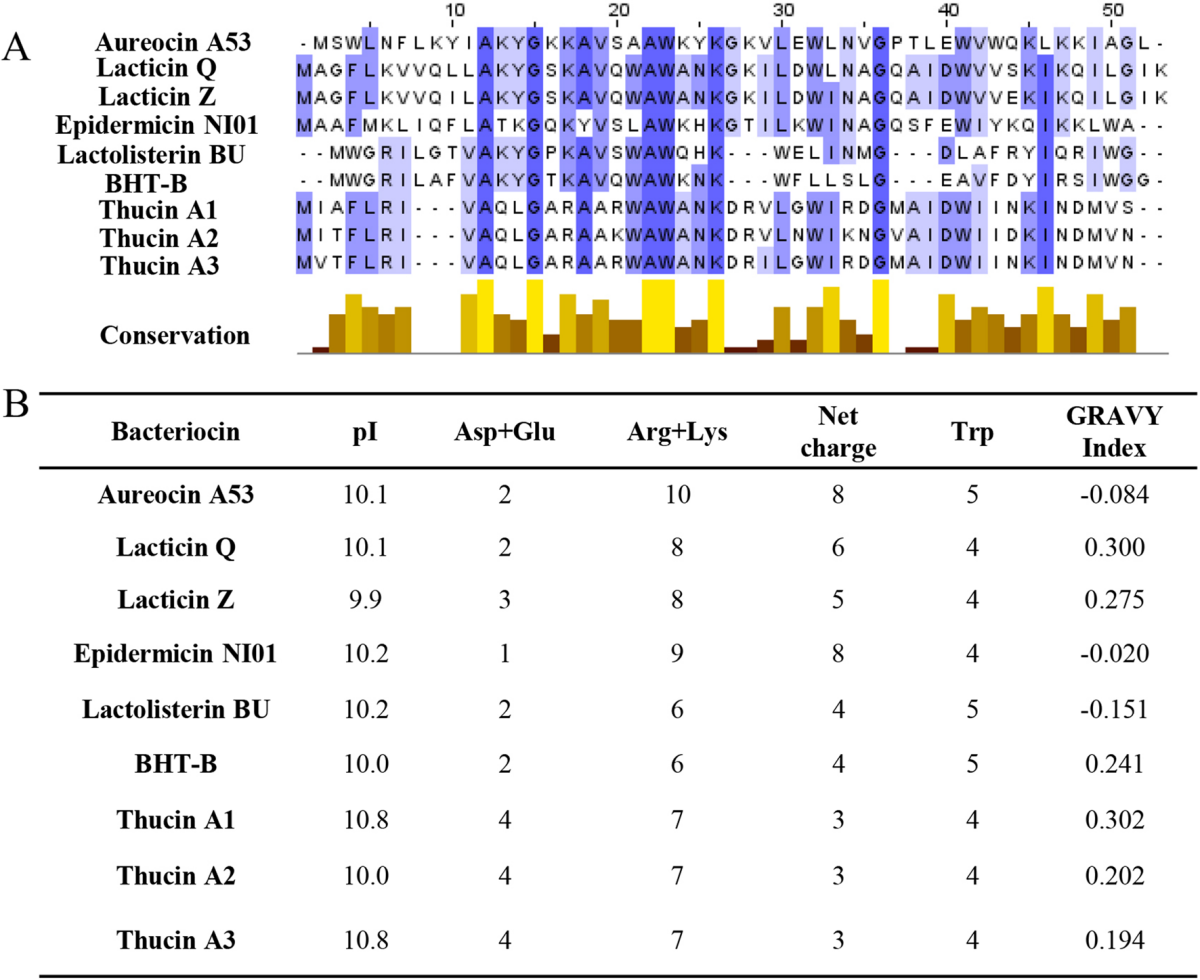
Sequence alignment (**A**) and putative relevant characteristics of Aur53-like leaderless bacteriocins (**B**)**.** Sequence alignment by Jalview 2.10.4b1; Putative relevant characteristics by ExPaSy ProtParam Tool; GRAVY, Grand average of hydropathicity

Sequence alignment analysis of thucin A1, thucin A2 and thucin A3 with known leaderless bacteriocins revealed that their amino acid sequences displayed high homology to those of aur53-like leaderless bacteriocins. In addition, they exhibited highly conserved N-terminal region (Ala_12_, Gly_15_, Ala_22_, Trp_23_ and Lys_26_) and relatively variable C-terminal region (Fig. 9A). Moreover, similar to aur53-like leaderless bacteriocins with high cationic charge, pI (isoelectric point), and Trp-rich peptides, each of thucin A1, thucin A2 and thucin A3 contained seven cationic charges and four Trp residues, and their predicted pI was 10.8, 10.0, 10.8, respectively (Fig. 9B). Previous studies have demonstrated that the Trp residues play a key role in the antimicrobial activity of aur53-like leaderless bacteriocins by facilitating their interaction with the bacterial membranes [46]. In addition, the electrostatic interaction with the negatively charged membrane and hydrophobic interaction also play a significant role in the initial binding to bacterial membranes [47]. Therefore, it can be speculated that the antimicrobial activity of thucin A1 may be related to its net charge and hydrophobicity.

We also tested the sensitivity of thucin A1 to pH, temperature and various proteases and found that thucin A1 has a wide acid–base adaptability (pH 2 to 11), even at a very low concentration (1× MIC). Moreover, thucin A1 exhibited extremely stable antimicrobial activity at pH 2 to 9 and lost some of its antimicrobial activity at pH 10 to 11; whereas nisin A was readily inactivated under neutral and alkaline conditions even at high concentrations (8× MIC) (Fig. 5B). Compared with nisin A, thucin A1 (4× MIC) showed good stability to trypsin and pepsin (Fig. 5E). In addition, both thucin A1 and nisin A could endure the temperature of 100 °C at concentrations above 2× MIC (Fig. 5C, )D). The stability of thucin A1 to pH, heat and enzyme indicates that it may be a promising alternative or supplement to nisin A in food industry.

In a previous study, the leaderless bacteriocin aureocin A53 was found to exert its bactericidal activity against sensitive strains by causing the leakage of vital molecules, dissipation of membrane potential, and termination of macromolecular synthesis [20]. In addition, the bactericidal activity of the leaderless bacteriocin toyoncin was attributed to the disruption of cell membranes [42]. In this study, we inferred that thucin A1 has bactericidal activity by monitoring the changes in the number of viable cells, the OD_600_ value and the amounts of K^+^ released in culture containing thucin A1 during incubation of the indictor bacteria.

When bacteriocins are used as a food biopreservative, the safety is of great importance. In previous studies, hemolytic activities have been detected in some bacteriocins, such as cytolysin and listeriolysin S [48, 49]. Therefore, the hemolytic activity and cytotoxicity of thucin A1 were measured to evaluate the safety of thucin A1. As a result, thucin A1 exhibited no hemolytic activity and cytotoxicity, even at concentrations much higher than its MIC values against a variety of indicator bacteria. In addition, recent studies have suggested that many leaderless bacteriocins, such as aureocin A53, aureocinA70 and toyoncin, can effectively inhibit or eliminate foodborne pathogens in skim milk, and possess desirable features to be used as food preservatives [1, 42, 50]. In the present study, we also observed that thucin A1 effectively inhibited or eliminated foodborne pathogens *B. cereus* and *L. monocytogenes* in skim milk. In the future, more detailed studies may be carried out about the safety of thucin A1 to evaluate the feasibility of its application as a biopreservative.

## Conclusion

In this study, three novel leaderless bacteriocins, thucin A1, A2 and A3, were identified in *B. thuringiensis* P86. As a representative, Thucin A1 exhibited stronger antibacterial activity against foodborne pathogens *B. cereus* and *L. monocytogenes* than nisin A, as well as certain activity against other food spoilage bacteria from air. In addition, thucin A1 showed wide acid–base adaptability, high endurance to heat, and good stability to trypsin and pepsin. More importantly, thucin A1 showed no hemolysis activity and cytotoxicity, and could effectively inhibit or eliminate two important foodborne pathogens in skim milk. These results indicate that these novel leadless bacteriocins may be promising alternative or supplement to nisin A as food biopreservative.

## Methods

### Screening of producers of broad-spectrum antimicrobial bacteriocins

A total of 100 *B. thuringiensis* strains isolated from soil samples in Henan Province in China, were tested for their antimicrobial activities against two important food pathogenic bacteria *B. cereus* ATCC 14579 and *L. monocytogenes* LM201 with the agar well diffusion method. These isolated strains were identified as *B. thuringiensis* by analyzing their 16S rRNA gene sequence and observing the forming of spore and the parasporal crystals. Briefly, each tested strain was incubated overnight in LB medium, and 1 mL culture was transferred into 100 mL of LB medium and incubated for 36 h at 30 °C and 220 rpm. The supernatant (10000×*g*, 3 min) was collected every 3 h from 3 to 36 h. In addition, the supernatant of the tested strain with a relatively large inhibition zone was treated with a mixed protease solution (trypsin, pepsin and proteinase K, the final concentration was 1 mg/mL). The strain that showed activity that was degraded by enzymes and showed the strongest antimicrobial activity against both two food pathogenic bacteria was chosen for further study.

### Antibacterial activity assay

Antibacterial activity against various indicator strains were determined by the agar well diffusion method [51]. Briefly, each of the indicator strains (10^6^ CFU/mL) was spread on a LB agar plate (45 °C) with wells (diameter = 6 mm). Then, 50 μL bacteriocin solution was added to the wells. The plate was kept at 4 °C for 2 h and subsequently incubated at a temperature suitable for the growth of the indicator strains for 12 h, followed by the measurement of inhibition zone to assess the antimicrobial activity.

### Genome sequencing and sequence analysis of *B. thuringiensis* P86

The genome of strain *B. thuringiensis* P86 was sequenced on an Illumina HiSeq 2500 platform (San Diego, CA, USA) to a final coverage of 150-fold. The genome assembly and annotation were performed with Abyss and Prokka. The bacteriocin biosynthetic gene clusters were predicted by BAGEL 4.0 [52]. Functional analysis of proteins was performed using the BLASTP program. Peptide or protein molecular mass was calculated by ProMACC (http://www.tofms.org/calmw/MyMWele.asp). Amino acid sequence alignment was performed using ClustalW [53].

### Purification of antimicrobial substances in strain *B. thuringiensis* P86

The *B. thuringiensis* P86 strain was grown overnight in LB medium. The activated culture was inoculated (1% v/v) in 2 L of LB medium at 30 °C with agitation at 220 rpm for 15 h. The fermentation culture was then centrifuged at 10000×*g* for 10 min at 4 °C. The antimicrobial crude extract was obtained using a previously described method [27]. Briefly, the cell-free supernatant was loaded onto a column containing 200 g of Amberlite XAD-7HP resin (Sigma, St. Louis, MO, USA). Then, the resin was washed with 2 L of ddH_2_O and 2 L of 30% (v/v) ethanol. The active substances were eluted in 1000 mL of 80% (v/v) ethanol (pH 2.0). The eluate was concentrated by a rotary evaporator and then lyophilized into powder (antimicrobial crude extract). The antimicrobial crude extract was dissolved in 5 mL of ddH_2_O and then subjected to HPLC analysis by Agilent TC-C18 column (4.6 × 250 mm, 5 μm) on a Shimadzu LC-20AT system. The mobile phase consisted of acetonitrile and HPLC-grade water containing 0.1% trifluoroacetic acid (TFA). About 30 μL of the sample was injected per run and separated by a linear gradient of 20–80% acetonitrile for 60 min at a flow rate of 1.0 mL/min. The eluate was monitored at a wavelength of 210 nm, and the fractions were manually collected for the bioassay of antibacterial activity against *B. cereus* ATCC 14579. The antibacterial fractions were collected and lyophilized into powder.

### LC–MS and LC–MS/MS analysis

Liquid chromatography-mass spectrometry (LC–MS) and LC–MS/MS analysis were performed by the Agilent 6540 Ultra High Definition (UHD) Accurate-Mass quadrupole time of flight (Q-TOF) LC–MS system to reveal the molecular mass of antibacterial fractions and analyze the structures of antibacterial fractions [54]. The analytical column was a ZORBAX Eclipse Plus C_18_ column (2.1 × 150 mm, 3.5 μm). The MS operating conditions were as follows: the flow rate of drying gas was 9 L/min, the temperature was 350 °C, the nebulizer pressure was 35 lb/in^2^, the capillary voltage was 3500 V, and the scanning range of Q-TOF MS was *m/z* 100–3000. Data were acquired at the rate of 1 spectrum/s. Detailed sequence information of the antimicrobial substances was further investigated with the targeted MS/MS mode. The target ion was isolated and fragmented by the application of a voltage of 40 V.

### Minimum inhibitory concentrations of bacteriocins

The minimum inhibitory concentrations (MICs) of the bacteriocins against various indicator strains were determined by a previously described method [51]. Briefly, the antimicrobial activities of different final concentrations bacteriocin solutions were measured by the agar well diffusion method described above. The MIC was defined at the lowest concentration of samples that could form a clear zone of inhibition.

### The stability assay of thucin A1 and nisin A to pH, temperature and protease

Nisin A was commercial nisin A (Sigma-Aldrich, Shanghai, China) purified by HPLC as described above. The purity of nisin A and thucin A1 was all greater than 95% based on the ratio of absorption peak area at 210 nm. The stability assay was performed by previously described method with some modifications [54]. To compare the pH stability of thucin A1 and nisin A, the bacteriocin solutions (1× , 2× , 4× , 8× MIC) were adjusted to pH 2.0, 3.0, 4.0, 5.0, 6.0, 7.0, 8.0, 9.0, 10.0, 11.0 and 12.0 with 0.5 M NaOH or 0.5 M HCl solution, followed by incubation at 37 °C for 4 h. The residual antimicrobial activity was tested after neutralizing the sample to pH 6.0. To compare the thermal stability of thucin A1 and nisin A, the bacteriocin solutions (1× , 2× , 4× , 8× MIC) were exposed to 37 °C, 50 °C, 60 °C, 70 °C, 80 °C, 90 °C and 100 °C for 2 h. The residual antimicrobial activity was tested. The effects of various proteases on thucin A1 and nisin A were also tested. The bacteriocin solution (4× MIC) and enzymes (1 mg/mL) were mixed at a 1:1 ratio (v/v) and incubated at 37 °C for 2 h, and the residual antimicrobial activity was determined. The proteases included trypsin (≥ 10,000 U/mg), pepsin (≥ 250 U/mg), and proteinase K (≥ 40 U/mg). The indicator strain used in this experiment was *B. cereus* ATCC 14579 and the antimicrobial activity was determined with the agar well diffusion method as described above. Untreated bacteriocin solutions were used as the control. All experiments were performed in triplicate.

### Bactericidal activity of thucin A1

Bactericidal activity of thucin A1 was evaluated using a previously described method [13]. Briefly, the purified thucin A1 at different concentrations (0, 1.88, 3.75, 7.5, 15 μM) was added to the culture of *B. cereus* ATCC14579 with an OD_600_ of ~ 0.5 and incubated at 30 °C for 3 h. The number of viable cells on LB agar plates and the OD_600_ were measured at different time points. The colonies were counted after incubation at 30 °C for 20 h and expressed as log CFU/mL.

### Potassium efflux assay

Potassium efflux assay was performed using a previously described method [42]. *Bacillus cereus* ATCC 14579 was incubated for 24 h at 30 °C 220 rpm and centrifuged at 3000 rpm for 10 min, the sedimented cells were washed three times with normal saline and resuspended in normal saline (OD600 = 0.5). Next, 1-ml amounts were incubated with thucin A1(0, 1.88, 3.75, 7.5, 15 μM) at 30 °C for different times (1, 2, and 3 h). The amounts of K^+^ released were tested by an Agilent 5100 ICP-MS (Agilent Technologies, Santa Clara, CA, USA).

### Hemolysis assay of thucin A1

The hemolysis assay of thucin A1 was performed using a previously described method with some modifications [5]. The hemolytic activity of thucin A1 was tested by measuring the release of hemoglobin from a suspension of defibrillated sheep blood (Shanghai yuanye Biotechnology Co., Ltd., China) at an absorbance of 540 nm. Briefly, defibrillated sheep blood was centrifuged at 3000 rpm for 10 min and the sedimented cells were washed three times with PBS buffer (pH 7.0). Then, 30 μL of re-suspended red blood cells were added to a 96-well plate containing 70 μL of thucin A1 at different final concentrations (3.75, 7.5, 15, 30, 60 and 120 μM) and incubated for 1 h at 37 °C. PBS buffer and Triton X-100 (1%) were used as negative and positive controls, respectively. After incubation, the samples were centrifuged at 3,000 rpm for 5 min, and the release of hemoglobin from the supernatant was tested at a wavelength of 540 nm. Hemolysis activity was calculated using the formula: hemolysis (%) = [(Abs_540_ of the bacteriocin treated sample)−(Abs_540_ of buffer treated sample)]/[(Abs_540_ of Triton X-100 treated sample)−(Abs_540_ of buffer treated sample)] × 100. All experiments were performed in triplicate.

### Cell toxicity assay of thucin A1

The cell toxicity assay of thucin A1 was performed using MTT [3-(4,5-dimethylthiazol-2-yl)-2,5-diphenyltetrazolium bromide] assay [55]. Briefly, HeLa cells were added a 96-well plate (~ 5 × 10^3^ cells / well) using Dulbecco’s modified Eagle’s medium (DMEM) supplemented with 10% fetal bovine serum and 1% penicillin–streptomycin. The cells were incubated at 37 °C in 5% CO_2_ for 24 h. The growth medium was replaced with fresh medium containing different concentrations of thucin A1 (3.75, 7.5, 15, 30 and 60 μM). The medium without thucin A1 was used as a negative control, and 1% Triton X-100 was used as a positive control. After the plate was incubated for 24 h, 20 μL of MTT solution (5 mg/ml in PBS, Solarbio, Shanghai, China) was added to the well, and then plates were incubated for 3 h at 37 °C. Then, the medium was removed and 50 μL dimethyl sulfoxide was added to the well. To assess the percentage of live cells in sample, absorbance (590 nm) was measured by Varioskan LUX Multimode Microplate Reader. The percentage of cell viability was calculated using the formula: cell viability (%) = 100−[(Abs_590_ of negative control sample)−(Abs_590_ of the bacteriocin treated sample)]/[(Abs_590_ of negative control sample)−(Abs_590_ of Triton X-100 treated sample)] × 100.

### Inhibition of foodborne pathogens in skim milk by thucin A1

To evaluate the antibacterial potential of thucin A1 in food, four sets of 5 mL available ultrahigh temperature (UHT) skim milk were inoculated with *B. cereus* ATCC14579 and *L. monocytogenes* LM201 at a concentration of 10^4^ CFU/mL, followed by the addition of thucin A1 at different final concentrations (0× , 8× and 16× MIC) and incubation at 25 °C for 7 days. Nisin A (1000 U) and ddH_2_O were used as positive and negative controls, respectively. The amount of viable cells in the milk samples was determined by plate counting.

### The statistical analysis

The statistical analyses were conducted by SPSS statistical software 20.0.

### Accession numbers

The nucleotide sequence of the thucin A gene cluster was deposited in the GenBank database under the accession number of ON398335.

## Supplementary Information

**Additional file 1: Fig. S1.** Proposed primary structure of thucin A2 and LC–MS/MS analysis of fraction A2. Fragment ions are indicated. “*” indicates that the N-terminal amino acid, methionine, was formylated.

**Additional file 2: Fig. S2.** Proposed primary structure of thucin A3 and LC–MS/MS analysis of fraction A3. Fragment ions are indicated. “*” indicates that the N-terminal amino acid, methionine, was formylated.

## Data Availability

All data generated or analyzed during this study are included in this published article.
