# Supplementary figures and images for: Three novel leaderless bacteriocins have antimicrobial activity against gram-positive bacteria to serve as promising food biopreservative
Source: Microb Cell Fact. 2022 Sep 19;21:194. doi: 10.1186/s12934-022-01912-3 (PMC9484092; doi:10.1186/s12934-022-01912-3)

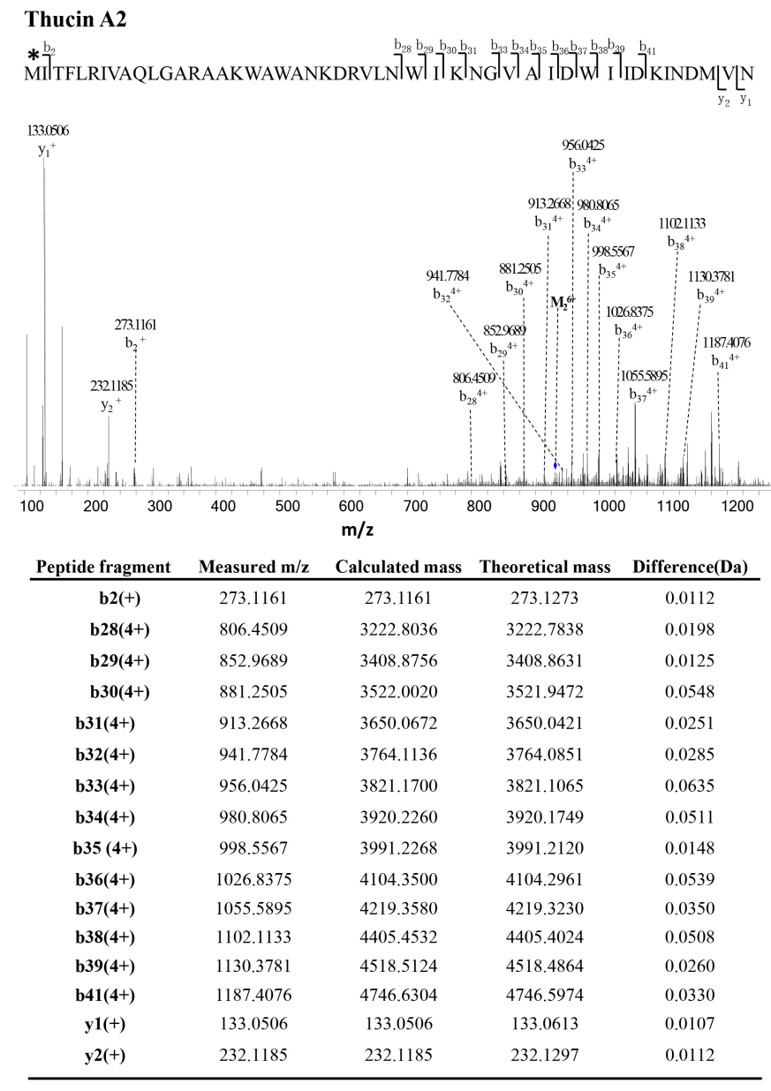

Supplement: Supplementary file 1 — Additional file 1: Fig. S1. Proposed primary structure of thucin A2 and LC–MS/MS analysis of fraction A2. Fragment ions are indicated. “*” indicates that the N-terminal amino acid, methionine, was formylated. [file 12934_2022_1912_MOESM1_ESM.jpg]

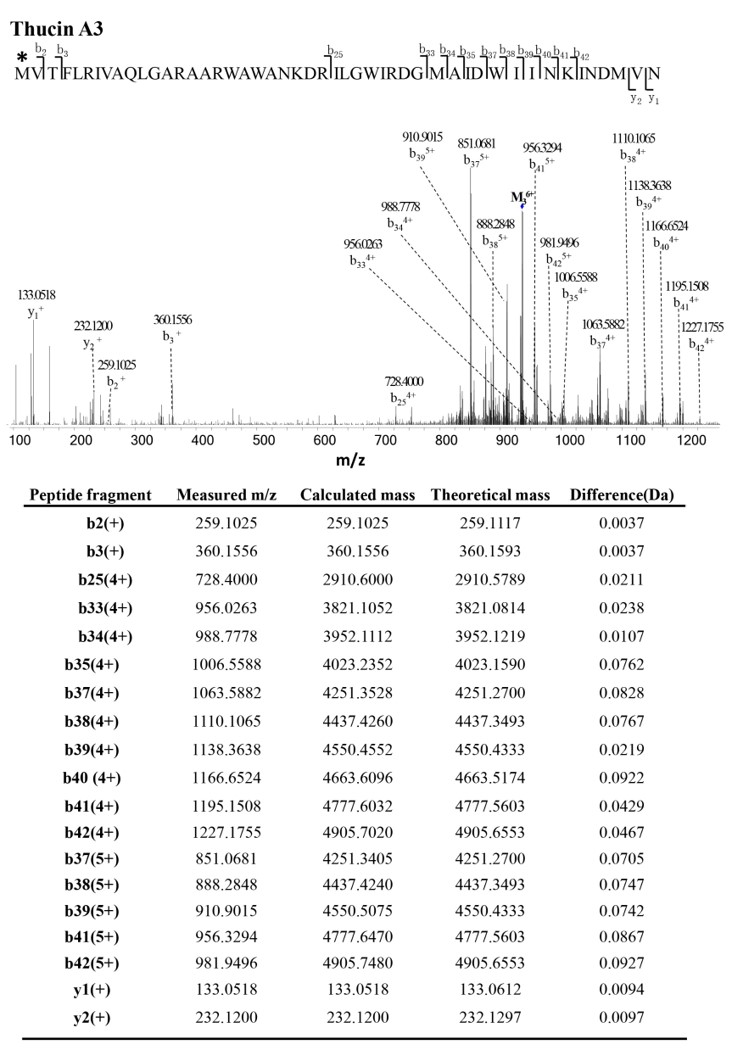

Supplement: Supplementary file 2 — Additional file 2: Fig. S2. Proposed primary structure of thucin A3 and LC–MS/MS analysis of fraction A3. Fragment ions are indicated. “*” indicates that the N-terminal amino acid, methionine, was formylated. [file 12934_2022_1912_MOESM2_ESM.jpg]
